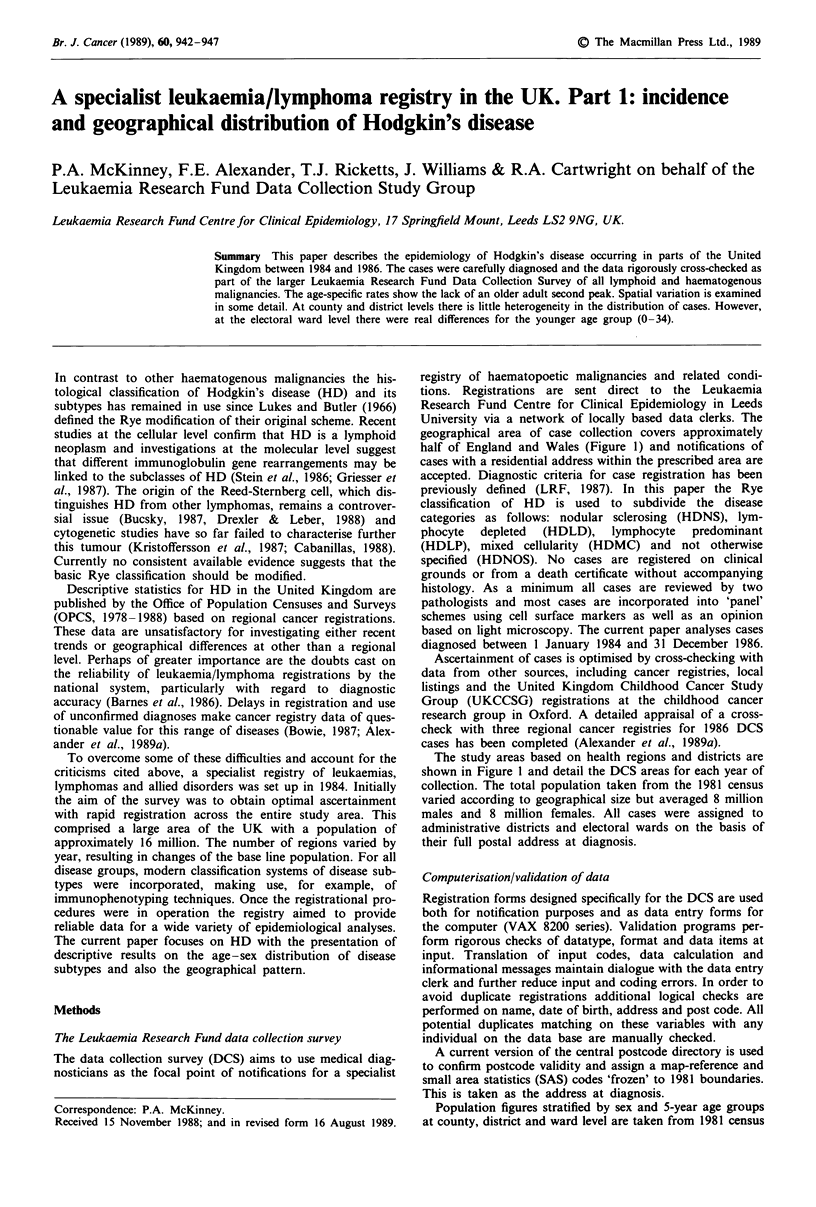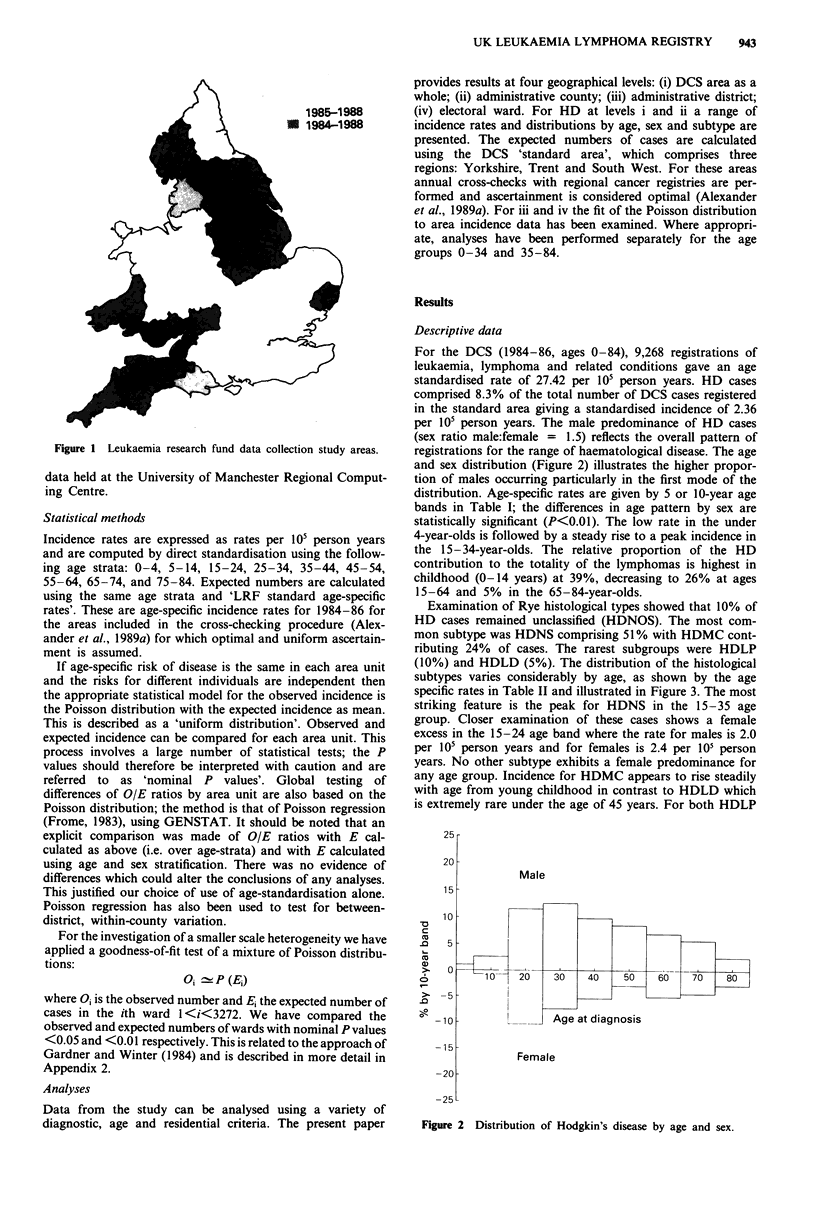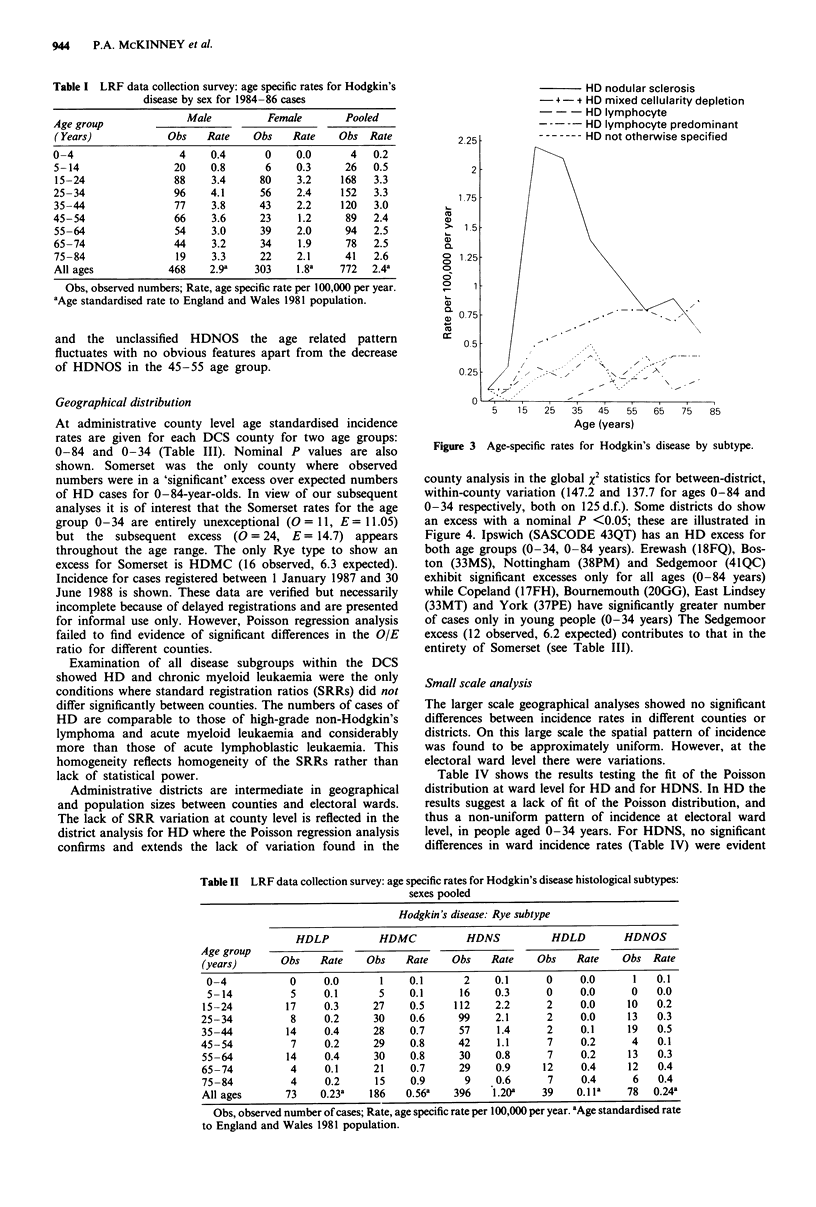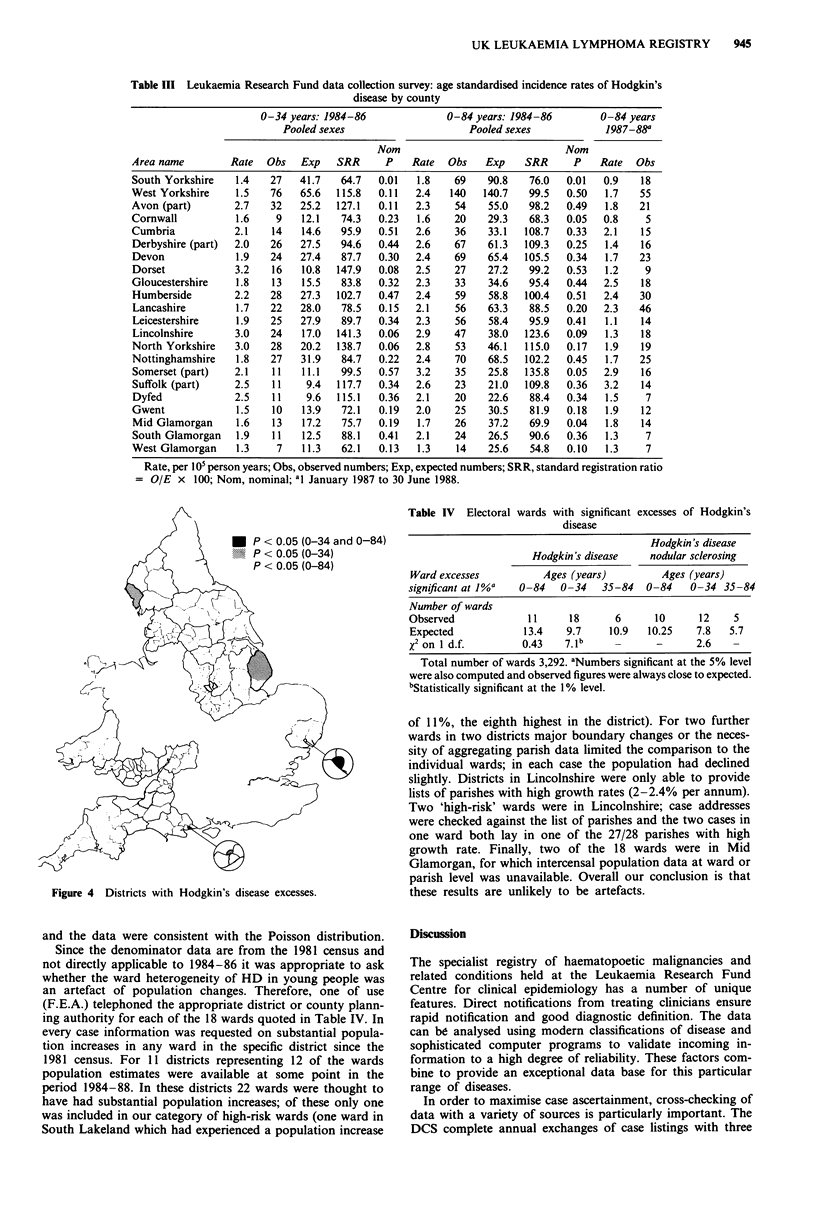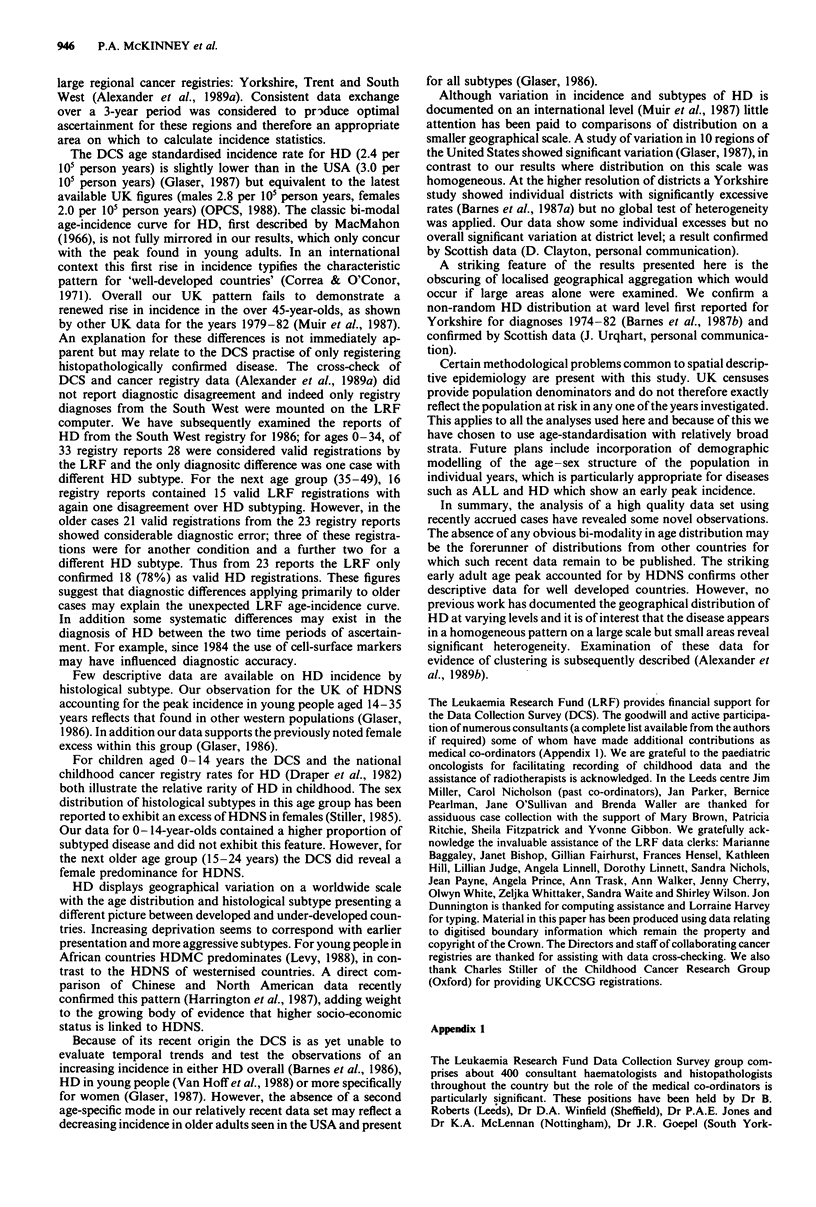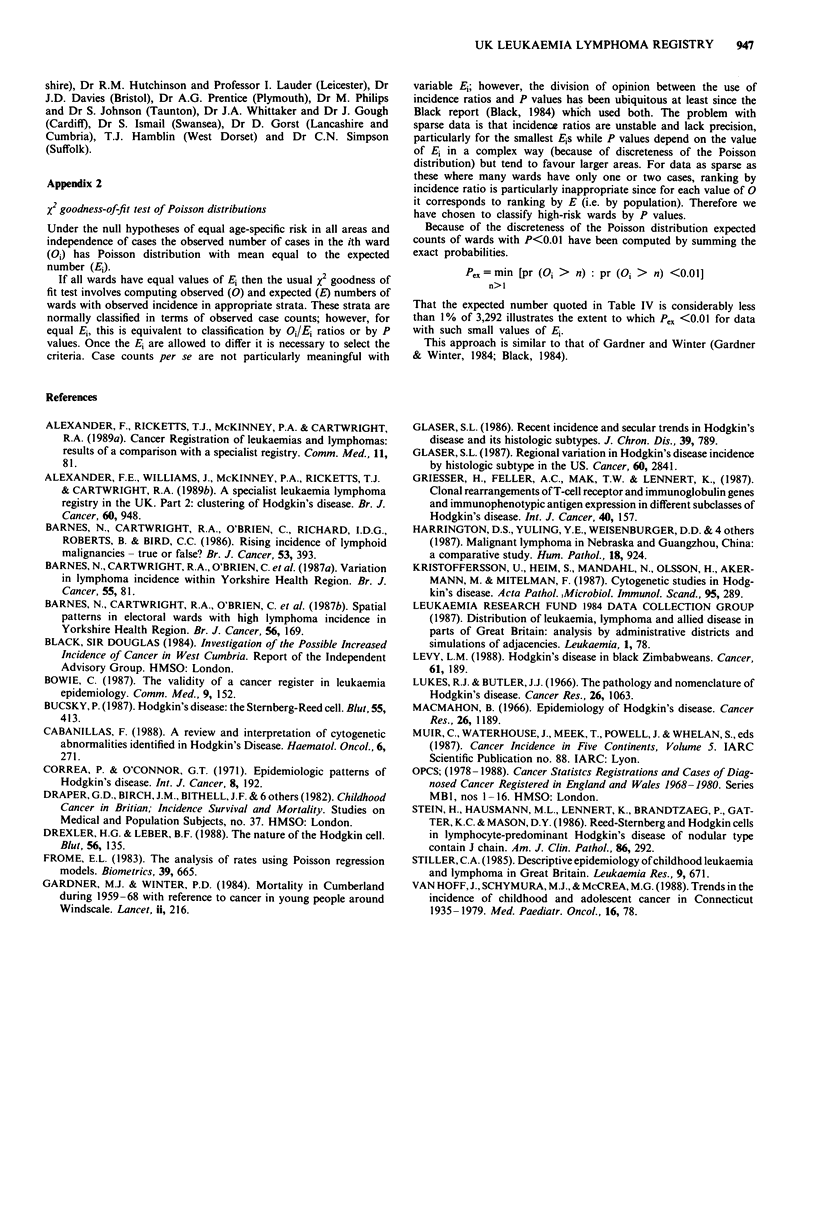# A specialist leukaemia/lymphoma registry in the UK. Part 1: Incidence and geographical distribution of Hodgkin's disease. Leukaemia Research Fund Data Collection Study Group.

**DOI:** 10.1038/bjc.1989.395

**Published:** 1989-12

**Authors:** P. A. McKinney, F. E. Alexander, T. J. Ricketts, J. Williams, R. A. Cartwright

**Affiliations:** Leukaemia Research Fund Centre for Clinical Epidemiology, Leeds, UK.

## Abstract

This paper describes the epidemiology of Hodgkin's disease occurring in parts of the United Kingdom between 1984 and 1986. The cases were carefully diagnosed and the data rigorously cross-checked as part of the larger Leukaemia Research Fund Data Collection Survey of all lymphoid and haematogenous malignancies. The age-specific rates show the lack of an older adult second peak. Spatial variation is examined in some detail. At county and district levels there is little heterogeneity in the distribution of cases. However, at the electoral ward level there were real differences for the younger age group (0-34).


*Br. J. Cancer* (1989), **60**, 942–947 © The Macmillan Press Ltd., 1989

### A specialist leukaemia/lymphoma registry in the UK. Part 1: incidence and geographical distribution of Hodgkin's disease

P.A. McKinney, F.E. Alexander, T.J. Ricketts, J. Williams & R.A. Cartwright on behalf of the Leukaemia Research Fund Data Collection Study Group

*Leukaemia Research Fund Centre for Clinical Epidemiology, 17 Springfield Mount, Leeds LS2 9NG, UK.*

**Summary** This paper describes the epidemiology of Hodgkin's disease occurring in parts of the United Kingdom between 1984 and 1986. The cases were carefully diagnosed and the data rigorously cross-checked as part of the larger Leukaemia Research Fund Data Collection Survey of all lymphoid and haematogenous malignancies. The age-specific rates show the lack of an older adult second peak. Spatial variation is examined in some detail. At county and district levels there is little heterogeneity in the distribution of cases. However, at the electoral ward level there were real differences for the younger age group (0–34).

In contrast to other haematogenous malignancies the histological classification of Hodgkin's disease (HD) and its subtypes has remained in use since Lukes and Butler (1966) defined the Rye modification of their original scheme. Recent studies at the cellular level confirm that HD is a lymphoid neoplasm and investigations at the molecular level suggest that different immunoglobulin gene rearrangements may be linked to the subclasses of HD (Stein *et al*., 1986; Griesser *et al*., 1987). The origin of the Reed-Sternberg cell, which distinguishes HD from other lymphomas, remains a controversial issue (Bucsky, 1987, Drexler & Leber, 1988) and cytogenetic studies have so far failed to characterise further this tumour (Kristoffersson *et al*., 1987; Cabanillas, 1988). Currently no consistent available evidence suggests that the basic Rye classification should be modified.

Descriptive statistics for HD in the United Kingdom are published by the Office of Population Censuses and Surveys (OPCS, 1978–1988) based on regional cancer registrations. These data are unsatisfactory for investigating either recent trends or geographical differences at other than a regional level. Perhaps of greater importance are the doubts cast on the reliability of leukaemia/lymphoma registrations by the national system, particularly with regard to diagnostic accuracy (Barnes *et al*., 1986). Delays in registration and use of unconfirmed diagnoses make cancer registry data of questionable value for this range of diseases (Bowie, 1987; Alexander *et al*., 1989*a*).

To overcome some of these difficulties and account for the criticisms cited above, a specialist registry of leukaemias, lymphomas and allied disorders was set up in 1984. Initially the aim of the survey was to obtain optimal ascertainment with rapid registration across the entire study area. This comprised a large area of the UK with a population of approximately 16 million. The number of regions varied by year, resulting in changes of the base line population. For all disease groups, modern classification systems of disease subtypes were incorporated, making use, for example, of immunophenotyping techniques. Once the registrational procedures were in operation the registry aimed to provide reliable data for a wide variety of epidemiological analyses. The current paper focuses on HD with the presentation of descriptive results on the age–sex distribution of disease subtypes and also the geographical pattern.

### Methods

### *The Leukaemia Research Fund data collection survey*

The data collection survey (DCS) aims to use medical diagnosticians as the focal point of notifications for a specialist registry of haematopoetic malignancies and related conditions. Registrations are sent direct to the Leukaemia Research Fund Centre for Clinical Epidemiology in Leeds University via a network of locally based data clerks. The geographical area of case collection covers approximately half of England and Wales (Figure 1) and notifications of cases with a residential address within the prescribed area are accepted. Diagnostic criteria for case registration has been previously defined (LRF, 1987). In this paper the Rye classification of HD is used to subdivide the disease categories as follows: nodular sclerosing (HDNS), lymphocyte depleted (HDLD), lymphocyte predominant (HDLP), mixed cellularity (HDMC) and not otherwise specified (HDNOS). No cases are registered on clinical grounds or from a death certificate without accompanying histology. As a minimum all cases are reviewed by two pathologists and most cases are incorporated into 'panel' schemes using cell surface markers as well as an opinion based on light microscopy. The current paper analyses cases diagnosed between 1 January 1984 and 31 December 1986.

Ascertainment of cases is optimised by cross-checking with data from other sources, including cancer registries, local listings and the United Kingdom Childhood Cancer Study Group (UKCCSG) registrations at the childhood cancer research group in Oxford. A detailed appraisal of a cross-check with three regional cancer registries for 1986 DCS cases has been completed (Alexander *et al*., 1989*a*).

The study areas based on health regions and districts are shown in Figure 1 and detail the DCS areas for each year of collection. The total population taken from the 1981 census varied according to geographical size but averaged 8 million males and 8 million females. All cases were assigned to administrative districts and electoral wards on the basis of their full postal address at diagnosis.

### *Computerisation/validation of data*

Registration forms designed specifically for the DCS are used both for notification purposes and as data entry forms for the computer (VAX 8200 series). Validation programs perform rigorous checks of datatype, format and data items at input. Translation of input codes, data calculation and informational messages maintain dialogue with the data entry clerk and further reduce input and coding errors. In order to avoid duplicate registrations additional logical checks are performed on name, date of birth, address and post code. All potential duplicates matching on these variables with any individual on the data base are manually checked.

A current version of the central postcode directory is used to confirm postcode validity and assign a map-reference and small area statistics (SAS) codes 'frozen' to 1981 boundaries. This is taken as the address at diagnosis.

Population figures stratified by sex and 5-year age groups at county, district and ward level are taken from 1981 census

Correspondence: P.A. McKinney.
Received 15 November 1988; and in revised form 16 August 1989.

**Figure 1** Leukaemia research fund data collection study areas.

data held at the University of Manchester Regional Computing Centre.

*Statistical methods*

Incidence rates are expressed as rates per $10^5$ person years and are computed by direct standardisation using the following age strata: 0–4, 5–14, 15–24, 25–34, 35–44, 45–54, 55–64, 65–74, and 75–84. Expected numbers are calculated using the same age strata and 'LRF standard age-specific rates'. These are age-specific incidence rates for 1984–86 for the areas included in the cross-checking procedure (Alexander *et al.*, 1989*a*) for which optimal and uniform ascertainment is assumed.

If age-specific risk of disease is the same in each area unit and the risks for different individuals are independent then the appropriate statistical model for the observed incidence is the Poisson distribution with the expected incidence as mean. This is described as a 'uniform distribution'. Observed and expected incidence can be compared for each area unit. This process involves a large number of statistical tests; the *P* values should therefore be interpreted with caution and are referred to as 'nominal *P* values'. Global testing of differences of $O/E$ ratios by area unit are also based on the Poisson distribution; the method is that of Poisson regression (Frome, 1983), using GENSTAT. It should be noted that an explicit comparison was made of $O/E$ ratios with $E$ calculated as above (i.e. over age-strata) and with $E$ calculated using age and sex stratification. There was no evidence of differences which could alter the conclusions of any analyses. This justified our choice of use of age-standardisation alone. Poisson regression has also been used to test for between-district, within-county variation.

For the investigation of a smaller scale heterogeneity we have applied a goodness-of-fit test of a mixture of Poisson distributions:

$$O_i \simeq P(E_i)$$

where $O_i$ is the observed number and $E_i$ the expected number of cases in the *i*th ward $1<i<3272$. We have compared the observed and expected numbers of wards with nominal *P* values $<0.05$ and $<0.01$ respectively. This is related to the approach of Gardner and Winter (1984) and is described in more detail in Appendix 2.

*Analyses*

Data from the study can be analysed using a variety of diagnostic, age and residential criteria. The present paper provides results at four geographical levels: (i) DCS area as a whole; (ii) administrative county; (iii) administrative district; (iv) electoral ward. For HD at levels i and ii a range of incidence rates and distributions by age, sex and subtype are presented. The expected numbers of cases are calculated using the DCS 'standard area', which comprises three regions: Yorkshire, Trent and South West. For these areas annual cross-checks with regional cancer registries are performed and ascertainment is considered optimal (Alexander *et al.*, 1989*a*). For iii and iv the fit of the Poisson distribution to area incidence data has been examined. Where appropriate, analyses have been performed separately for the age groups 0–34 and 35–84.

### Results

*Descriptive data*

For the DCS (1984–86, ages 0–84), 9,268 registrations of leukaemia, lymphoma and related conditions gave an age standardised rate of 27.42 per $10^5$ person years. HD cases comprised 8.3% of the total number of DCS cases registered in the standard area giving a standardised incidence of 2.36 per $10^5$ person years. The male predominance of HD cases (sex ratio male:female = 1.5) reflects the overall pattern of registrations for the range of haematological disease. The age and sex distribution (Figure 2) illustrates the higher proportion of males occurring particularly in the first mode of the distribution. Age-specific rates are given by 5 or 10-year age bands in Table I; the differences in age pattern by sex are statistically significant ($P<0.01$). The low rate in the under 4-year-olds is followed by a steady rise to a peak incidence in the 15–34-year-olds. The relative proportion of the HD contribution to the totality of the lymphomas is highest in childhood (0–14 years) at 39%, decreasing to 26% at ages 15–64 and 5% in the 65–84-year-olds.

Examination of Rye histological types showed that 10% of HD cases remained unclassified (HDNOS). The most common subtype was HDNS comprising 51% with HDMC contributing 24% of cases. The rarest subgroups were HDLP (10%) and HDLD (5%). The distribution of the histological subtypes varies considerably by age, as shown by the age specific rates in Table II and illustrated in Figure 3. The most striking feature is the peak for HDNS in the 15–35 age group. Closer examination of these cases shows a female excess in the 15–24 age band where the rate for males is 2.0 per $10^5$ person years and for females is 2.4 per $10^5$ person years. No other subtype exhibits a female predominance for any age group. Incidence for HDMC appears to rise steadily with age from young childhood in contrast to HDLD which is extremely rare under the age of 45 years. For both HDLP

**Figure 2** Distribution of Hodgkin's disease by age and sex.

**Table I** LRF data collection survey: age specific rates for Hodgkin's disease by sex for 1984–86 cases

| Age group (Years) | Male | | Female | | Pooled | |
|---|---|---|---|---|---|---|
| | Obs | Rate | Obs | Rate | Obs | Rate |
| 0–4 | 4 | 0.4 | 0 | 0.0 | 4 | 0.2 |
| 5–14 | 20 | 0.8 | 6 | 0.3 | 26 | 0.5 |
| 15–24 | 88 | 3.4 | 80 | 3.2 | 168 | 3.3 |
| 25–34 | 96 | 4.1 | 56 | 2.4 | 152 | 3.3 |
| 35–44 | 77 | 3.8 | 43 | 2.2 | 120 | 3.0 |
| 45–54 | 66 | 3.6 | 23 | 1.2 | 89 | 2.4 |
| 55–64 | 54 | 3.0 | 39 | 2.0 | 94 | 2.5 |
| 65–74 | 44 | 3.2 | 34 | 1.9 | 78 | 2.5 |
| 75–84 | 19 | 3.3 | 22 | 2.1 | 41 | 2.6 |
| All ages | 468 | 2.9[a] | 303 | 1.8[a] | 772 | 2.4[a] |

Obs, observed numbers; Rate, age specific rate per 100,000 per year. [a]Age standardised rate to England and Wales 1981 population.

**Figure 3** Age-specific rates for Hodgkin's disease by subtype.

and the unclassified HDNOS the age related pattern fluctuates with no obvious features apart from the decrease of HDNOS in the 45–55 age group.

*Geographical distribution*

At administrative county level age standardised incidence rates are given for each DCS county for two age groups: 0–84 and 0–34 (Table III). Nominal $P$ values are also shown. Somerset was the only county where observed numbers were in a 'significant' excess over expected numbers of HD cases for 0–84-year-olds. In view of our subsequent analyses it is of interest that the Somerset rates for the age group 0–34 are entirely unexceptional ($O = 11$, $E = 11.05$) but the subsequent excess ($O = 24$, $E = 14.7$) appears throughout the age range. The only Rye type to show an excess for Somerset is HDMC (16 observed, 6.3 expected). Incidence for cases registered between 1 January 1987 and 30 June 1988 is shown. These data are verified but necessarily incomplete because of delayed registrations and are presented for informal use only. However, Poisson regression analysis failed to find evidence of significant differences in the $O/E$ ratio for different counties.

Examination of all disease subgroups within the DCS showed HD and chronic myeloid leukaemia were the only conditions where standard registration ratios (SRRs) did *not* differ significantly between counties. The numbers of cases of HD are comparable to those of high-grade non-Hodgkin's lymphoma and acute myeloid leukaemia and considerably more than those of acute lymphoblastic leukaemia. This homogeneity reflects homogeneity of the SRRs rather than lack of statistical power.

Administrative districts are intermediate in geographical and population sizes between counties and electoral wards. The lack of SRR variation at county level is reflected in the district analysis for HD where the Poisson regression analysis confirms and extends the lack of variation found in the county analysis in the global $\chi^2$ statistics for between-district, within-county variation (147.2 and 137.7 for ages 0–84 and 0–34 respectively, both on 125 d.f.). Some districts do show an excess with a nominal $P < 0.05$; these are illustrated in Figure 4. Ipswich (SASCODE 43QT) has an HD excess for both age groups (0–34, 0–84 years). Erewash (18FQ), Boston (33MS), Nottingham (38PM) and Sedgemoor (41QC) exhibit significant excesses only for all ages (0–84 years) while Copeland (17FH), Bournemouth (20GG), East Lindsey (33MT) and York (37PE) have significantly greater number of cases only in young people (0–34 years) The Sedgemoor excess (12 observed, 6.2 expected) contributes to that in the entirety of Somerset (see Table III).

*Small scale analysis*

The larger scale geographical analyses showed no significant differences between incidence rates in different counties or districts. On this large scale the spatial pattern of incidence was found to be approximately uniform. However, at the electoral ward level there were variations.

Table IV shows the results testing the fit of the Poisson distribution at ward level for HD and for HDNS. In HD the results suggest a lack of fit of the Poisson distribution, and thus a non-uniform pattern of incidence at electoral ward level, in people aged 0–34 years. For HDNS, no significant differences in ward incidence rates (Table IV) were evident

**Table II** LRF data collection survey: age specific rates for Hodgkin's disease histological subtypes: sexes pooled

| Age group (years) | Hodgkin's disease: Rye subtype | | | | | | | | | |
|---|---|---|---|---|---|---|---|---|---|---|
| | HDLP | | HDMC | | HDNS | | HDLD | | HDNOS | |
| | Obs | Rate | Obs | Rate | Obs | Rate | Obs | Rate | Obs | Rate |
| 0–4 | 0 | 0.0 | 1 | 0.1 | 2 | 0.1 | 0 | 0.0 | 1 | 0.1 |
| 5–14 | 5 | 0.1 | 5 | 0.1 | 16 | 0.3 | 0 | 0.0 | 0 | 0.0 |
| 15–24 | 17 | 0.3 | 27 | 0.5 | 112 | 2.2 | 2 | 0.0 | 10 | 0.2 |
| 25–34 | 8 | 0.2 | 30 | 0.6 | 99 | 2.1 | 2 | 0.0 | 13 | 0.3 |
| 35–44 | 14 | 0.4 | 28 | 0.7 | 57 | 1.4 | 2 | 0.1 | 19 | 0.5 |
| 45–54 | 7 | 0.2 | 29 | 0.8 | 42 | 1.1 | 7 | 0.2 | 4 | 0.1 |
| 55–64 | 14 | 0.4 | 30 | 0.8 | 30 | 0.8 | 7 | 0.2 | 13 | 0.3 |
| 65–74 | 4 | 0.1 | 21 | 0.7 | 29 | 0.9 | 12 | 0.4 | 12 | 0.4 |
| 75–84 | 4 | 0.2 | 15 | 0.9 | 9 | 0.6 | 7 | 0.4 | 6 | 0.4 |
| All ages | 73 | 0.23[a] | 186 | 0.56[a] | 396 | 1.20[a] | 39 | 0.11[a] | 78 | 0.24[a] |

Obs, observed number of cases; Rate, age specific rate per 100,000 per year. [a]Age standardised rate to England and Wales 1981 population.

**Table III** Leukaemia Research Fund data collection survey: age standardised incidence rates of Hodgkin's disease by county

| | *0–34 years: 1984–86 Pooled sexes* | | | | | *0–84 years: 1984–86 Pooled sexes* | | | | | *0–84 years 1987–88[a]* | |
|---|---|---|---|---|---|---|---|---|---|---|---|---|
| *Area name* | *Rate* | *Obs* | *Exp* | *SRR* | *Nom P* | *Rate* | *Obs* | *Exp* | *SRR* | *Nom P* | *Rate* | *Obs* |
| South Yorkshire | 1.4 | 27 | 41.7 | 64.7 | 0.01 | 1.8 | 69 | 90.8 | 76.0 | 0.01 | 0.9 | 18 |
| West Yorkshire | 1.5 | 76 | 65.6 | 115.8 | 0.11 | 2.4 | 140 | 140.7 | 99.5 | 0.50 | 1.7 | 55 |
| Avon (part) | 2.7 | 32 | 25.2 | 127.1 | 0.11 | 2.3 | 54 | 55.0 | 98.2 | 0.49 | 1.8 | 21 |
| Cornwall | 1.6 | 9 | 12.1 | 74.3 | 0.23 | 1.6 | 20 | 29.3 | 68.3 | 0.05 | 0.8 | 5 |
| Cumbria | 2.1 | 14 | 14.6 | 95.9 | 0.51 | 2.6 | 36 | 33.1 | 108.7 | 0.33 | 2.1 | 15 |
| Derbyshire (part) | 2.0 | 26 | 27.5 | 94.6 | 0.44 | 2.6 | 67 | 61.3 | 109.3 | 0.25 | 1.4 | 16 |
| Devon | 1.9 | 24 | 27.4 | 87.7 | 0.30 | 2.4 | 69 | 65.4 | 105.5 | 0.34 | 1.7 | 23 |
| Dorset | 3.2 | 16 | 10.8 | 147.9 | 0.08 | 2.5 | 27 | 27.2 | 99.2 | 0.53 | 1.2 | 9 |
| Gloucestershire | 1.8 | 13 | 15.5 | 83.8 | 0.32 | 2.3 | 33 | 34.6 | 95.4 | 0.44 | 2.5 | 18 |
| Humberside | 2.2 | 28 | 27.3 | 102.7 | 0.47 | 2.4 | 59 | 58.8 | 100.4 | 0.51 | 2.4 | 30 |
| Lancashire | 1.7 | 22 | 28.0 | 78.5 | 0.15 | 2.1 | 56 | 63.3 | 88.5 | 0.20 | 2.3 | 46 |
| Leicestershire | 1.9 | 25 | 27.9 | 89.7 | 0.34 | 2.3 | 56 | 58.4 | 95.9 | 0.41 | 1.1 | 14 |
| Lincolnshire | 3.0 | 24 | 17.0 | 141.3 | 0.06 | 2.9 | 47 | 38.0 | 123.6 | 0.09 | 1.3 | 18 |
| North Yorkshire | 3.0 | 28 | 20.2 | 138.7 | 0.06 | 2.8 | 53 | 46.1 | 115.0 | 0.17 | 1.9 | 19 |
| Nottinghamshire | 1.8 | 27 | 31.9 | 84.7 | 0.22 | 2.4 | 70 | 68.5 | 102.2 | 0.45 | 1.7 | 25 |
| Somerset (part) | 2.1 | 11 | 11.1 | 99.5 | 0.57 | 3.2 | 35 | 25.8 | 135.8 | 0.05 | 2.9 | 16 |
| Suffolk (part) | 2.5 | 11 | 9.4 | 117.7 | 0.34 | 2.6 | 23 | 21.0 | 109.8 | 0.36 | 3.2 | 14 |
| Dyfed | 2.5 | 11 | 9.6 | 115.1 | 0.36 | 2.1 | 20 | 22.6 | 88.4 | 0.34 | 1.5 | 7 |
| Gwent | 1.5 | 10 | 13.9 | 72.1 | 0.19 | 2.0 | 25 | 30.5 | 81.9 | 0.18 | 1.9 | 12 |
| Mid Glamorgan | 1.6 | 13 | 17.2 | 75.7 | 0.19 | 1.7 | 26 | 37.2 | 69.9 | 0.04 | 1.8 | 14 |
| South Glamorgan | 1.9 | 11 | 12.5 | 88.1 | 0.41 | 2.1 | 24 | 26.5 | 90.6 | 0.36 | 1.3 | 7 |
| West Glamorgan | 1.3 | 7 | 11.3 | 62.1 | 0.13 | 1.3 | 14 | 25.6 | 54.8 | 0.10 | 1.3 | 7 |

Rate, per $10^5$ person years; Obs, observed numbers; Exp, expected numbers; SRR, standard registration ratio $= O/E \times 100$; Nom, nominal; [a]1 January 1987 to 30 June 1988.

**Figure 4** Districts with Hodgkin's disease excesses.

**Table IV** Electoral wards with significant excesses of Hodgkin's disease

| | *Hodgkin's disease* | | | *Hodgkin's disease nodular sclerosing* | | |
|---|---|---|---|---|---|---|
| *Ward excesses significant at 1%[a]* | *Ages (years) 0–84* | *0–34* | *35–84* | *Ages (years) 0–84* | *0–34* | *35–84* |
| *Number of wards* | | | | | | |
| Observed | 11 | 18 | 6 | 10 | 12 | 5 |
| Expected | 13.4 | 9.7 | 10.9 | 10.25 | 7.8 | 5.7 |
| $\chi^2$ on 1 d.f. | 0.43 | 7.1[b] | – | – | 2.6 | – |

Total number of wards 3,292. [a]Numbers significant at the 5% level were also computed and observed figures were always close to expected. [b]Statistically significant at the 1% level.

and the data were consistent with the Poisson distribution.

Since the denominator data are from the 1981 census and not directly applicable to 1984–86 it was appropriate to ask whether the ward heterogeneity of HD in young people was an artefact of population changes. Therefore, one of use (F.E.A.) telephoned the appropriate district or county planning authority for each of the 18 wards quoted in Table IV. In every case information was requested on substantial population increases in any ward in the specific district since the 1981 census. For 11 districts representing 12 of the wards population estimates were available at some point in the period 1984–88. In these districts 22 wards were thought to have had substantial population increases; of these only one was included in our category of high-risk wards (one ward in South Lakeland which had experienced a population increase of 11%, the eighth highest in the district). For two further wards in two districts major boundary changes or the necessity of aggregating parish data limited the comparison to the individual wards; in each case the population had declined slightly. Districts in Lincolnshire were only able to provide lists of parishes with high growth rates (2–2.4% per annum). Two 'high-risk' wards were in Lincolnshire; case addresses were checked against the list of parishes and the two cases in one ward both lay in one of the 27/28 parishes with high growth rate. Finally, two of the 18 wards were in Mid Glamorgan, for which intercensal population data at ward or parish level was unavailable. Overall our conclusion is that these results are unlikely to be artefacts.

### Discussion

The specialist registry of haematopoetic malignancies and related conditions held at the Leukaemia Research Fund Centre for clinical epidemiology has a number of unique features. Direct notifications from treating clinicians ensure rapid notification and good diagnostic definition. The data can bé analysed using modern classifications of disease and sophisticated computer programs to validate incoming information to a high degree of reliability. These factors combine to provide an exceptional data base for this particular range of diseases.

In order to maximise case ascertainment, cross-checking of data with a variety of sources is particularly important. The DCS complete annual exchanges of case listings with three large regional cancer registries: Yorkshire, Trent and South West (Alexander *et al.*, 1989*a*). Consistent data exchange over a 3-year period was considered to produce optimal ascertainment for these regions and therefore an appropriate area on which to calculate incidence statistics.

The DCS age standardised incidence rate for HD (2.4 per $10^5$ person years) is slightly lower than in the USA (3.0 per $10^5$ person years) (Glaser, 1987) but equivalent to the latest available UK figures (males 2.8 per $10^5$ person years, females 2.0 per $10^5$ person years) (OPCS, 1988). The classic bi-modal age-incidence curve for HD, first described by MacMahon (1966), is not fully mirrored in our results, which only concur with the peak found in young adults. In an international context this first rise in incidence typifies the characteristic pattern for 'well-developed countries' (Correa & O'Conor, 1971). Overall our UK pattern fails to demonstrate a renewed rise in incidence in the over 45-year-olds, as shown by other UK data for the years 1979–82 (Muir *et al.*, 1987). An explanation for these differences is not immediately apparent but may relate to the DCS practise of only registering histopathologically confirmed disease. The cross-check of DCS and cancer registry data (Alexander *et al.*, 1989*a*) did not report diagnostic disagreement and indeed only registry diagnoses from the South West were mounted on the LRF computer. We have subsequently examined the reports of HD from the South West registry for 1986; for ages 0–34, of 33 registry reports 28 were considered valid registrations by the LRF and the only diagnositc difference was one case with different HD subtype. For the next age group (35–49), 16 registry reports contained 15 valid LRF registrations with again one disagreement over HD subtyping. However, in the older cases 21 valid registrations from the 23 registry reports showed considerable diagnostic error; three of these registrations were for another condition and a further two for a different HD subtype. Thus from 23 reports the LRF only confirmed 18 (78%) as valid HD registrations. These figures suggest that diagnostic differences applying primarily to older cases may explain the unexpected LRF age-incidence curve. In addition some systematic differences may exist in the diagnosis of HD between the two time periods of ascertainment. For example, since 1984 the use of cell-surface markers may have influenced diagnostic accuracy.

Few descriptive data are available on HD incidence by histological subtype. Our observation for the UK of HDNS accounting for the peak incidence in young people aged 14–35 years reflects that found in other western populations (Glaser, 1986). In addition our data supports the previously noted female excess within this group (Glaser, 1986).

For children aged 0–14 years the DCS and the national childhood cancer registry rates for HD (Draper *et al.*, 1982) both illustrate the relative rarity of HD in childhood. The sex distribution of histological subtypes in this age group has been reported to exhibit an excess of HDNS in females (Stiller, 1985). Our data for 0–14-year-olds contained a higher proportion of subtyped disease and did not exhibit this feature. However, for the next older age group (15–24 years) the DCS did reveal a female predominance for HDNS.

HD displays geographical variation on a worldwide scale with the age distribution and histological subtype presenting a different picture between developed and under-developed countries. Increasing deprivation seems to correspond with earlier presentation and more aggressive subtypes. For young people in African countries HDMC predominates (Levy, 1988), in contrast to the HDNS of westernised countries. A direct comparison of Chinese and North American data recently confirmed this pattern (Harrington *et al.*, 1987), adding weight to the growing body of evidence that higher socio-economic status is linked to HDNS.

Because of its recent origin the DCS is as yet unable to evaluate temporal trends and test the observations of an increasing incidence in either HD overall (Barnes *et al.*, 1986), HD in young people (Van Hoff *et al.*, 1988) or more specifically for women (Glaser, 1987). However, the absence of a second age-specific mode in our relatively recent data set may reflect a decreasing incidence in older adults seen in the USA and present for all subtypes (Glaser, 1986).

Although variation in incidence and subtypes of HD is documented on an international level (Muir *et al.*, 1987) little attention has been paid to comparisons of distribution on a smaller geographical scale. A study of variation in 10 regions of the United States showed significant variation (Glaser, 1987), in contrast to our results where distribution on this scale was homogeneous. At the higher resolution of districts a Yorkshire study showed individual districts with significantly excessive rates (Barnes *et al.*, 1987*a*) but no global test of heterogeneity was applied. Our data show some individual excesses but no overall significant variation at district level; a result confirmed by Scottish data (D. Clayton, personal communication).

A striking feature of the results presented here is the obscuring of localised geographical aggregation which would occur if large areas alone were examined. We confirm a non-random HD distribution at ward level first reported for Yorkshire for diagnoses 1974–82 (Barnes *et al.*, 1987*b*) and confirmed by Scottish data (J. Urqhart, personal communication).

Certain methodological problems common to spatial descriptive epidemiology are present with this study. UK censuses provide population denominators and do not therefore exactly reflect the population at risk in any one of the years investigated. This applies to all the analyses used here and because of this we have chosen to use age-standardisation with relatively broad strata. Future plans include incorporation of demographic modelling of the age–sex structure of the population in individual years, which is particularly appropriate for diseases such as ALL and HD which show an early peak incidence.

In summary, the analysis of a high quality data set using recently accrued cases have revealed some novel observations. The absence of any obvious bi-modality in age distribution may be the forerunner of distributions from other countries for which such recent data remain to be published. The striking early adult age peak accounted for by HDNS confirms other descriptive data for well developed countries. However, no previous work has documented the geographical distribution of HD at varying levels and it is of interest that the disease appears in a homogeneous pattern on a large scale but small areas reveal significant heterogeneity. Examination of these data for evidence of clustering is subsequently described (Alexander *et al.*, 1989*b*).

The Leukaemia Research Fund (LRF) provides financial support for the Data Collection Survey (DCS). The goodwill and active participation of numerous consultants (a complete list available from the authors if required) some of whom have made additional contributions as medical co-ordinators (Appendix 1). We are grateful to the paediatric oncologists for facilitating recording of childhood data and the assistance of radiotherapists is acknowledged. In the Leeds centre Jim Miller, Carol Nicholson (past co-ordinators), Jan Parker, Bernice Pearlman, Jane O'Sullivan and Brenda Waller are thanked for assiduous case collection with the support of Mary Brown, Patricia Ritchie, Sheila Fitzpatrick and Yvonne Gibbon. We gratefully acknowledge the invaluable assistance of the LRF data clerks: Marianne Baggaley, Janet Bishop, Gillian Fairhurst, Frances Hensel, Kathleen Hill, Lillian Judge, Angela Linnell, Dorothy Linnett, Sandra Nichols, Jean Payne, Angela Prince, Ann Trask, Ann Walker, Jenny Cherry, Olwyn White, Zeljka Whittaker, Sandra Waite and Shirley Wilson. Jon Dunnington is thanked for computing assistance and Lorraine Harvey for typing. Material in this paper has been produced using data relating to digitised boundary information which remain the property and copyright of the Crown. The Directors and staff of collaborating cancer registries are thanked for assisting with data cross-checking. We also thank Charles Stiller of the Childhood Cancer Research Group (Oxford) for providing UKCCSG registrations.

**Appendix 1**

The Leukaemia Research Fund Data Collection Survey group comprises about 400 consultant haematologists and histopathologists throughout the country but the role of the medical co-ordinators is particularly significant. These positions have been held by Dr B. Roberts (Leeds), Dr D.A. Winfield (Sheffield), Dr P.A.E. Jones and Dr K.A. McLennan (Nottingham), Dr J.R. Goepel (South Yorkshire), Dr R.M. Hutchinson and Professor I. Lauder (Leicester), Dr J.D. Davies (Bristol), Dr A.G. Prentice (Plymouth), Dr M. Philips and Dr S. Johnson (Taunton), Dr J.A. Whittaker and Dr J. Gough (Cardiff), Dr S. Ismail (Swansea), Dr D. Gorst (Lancashire and Cumbria), T.J. Hamblin (West Dorset) and Dr C.N. Simpson (Suffolk).

### Appendix 2

*$\chi^2$ goodness-of-fit test of Poisson distributions*

Under the null hypotheses of equal age-specific risk in all areas and independence of cases the observed number of cases in the *i*th ward ($O_i$) has Poisson distribution with mean equal to the expected number ($E_i$).

If all wards have equal values of $E_i$ then the usual $\chi^2$ goodness of fit test involves computing observed ($O$) and expected ($E$) numbers of wards with observed incidence in appropriate strata. These strata are normally classified in terms of observed case counts; however, for equal $E_i$, this is equivalent to classification by $O_i/E_i$ ratios or by $P$ values. Once the $E_i$ are allowed to differ it is necessary to select the criteria. Case counts *per se* are not particularly meaningful with variable $E_i$; however, the division of opinion between the use of incidence ratios and $P$ values has been ubiquitous at least since the Black report (Black, 1984) which used both. The problem with sparse data is that incidence ratios are unstable and lack precision, particularly for the smallest $E_i$s while $P$ values depend on the value of $E_i$ in a complex way (because of discreteness of the Poisson distribution) but tend to favour larger areas. For data as sparse as these where many wards have only one or two cases, ranking by incidence ratio is particularly inappropriate since for each value of $O$ it corresponds to ranking by $E$ (i.e. by population). Therefore we have chosen to classify high-risk wards by $P$ values.

Because of the discreteness of the Poisson distribution expected counts of wards with $P<0.01$ have been computed by summing the exact probabilities.

$$P_{ex} = \min_{n>1} [\text{pr } (O_i > n) : \text{pr } (O_i > n) < 0.01]$$

That the expected number quoted in Table IV is considerably less than 1% of 3,292 illustrates the extent to which $P_{ex} < 0.01$ for data with such small values of $E_i$.

This approach is similar to that of Gardner and Winter (Gardner & Winter, 1984; Black, 1984).

### References

ALEXANDER, F., RICKETTS, T.J., McKINNEY, P.A. & CARTWRIGHT, R.A. (1989*a*). Cancer Registration of leukaemias and lymphomas: results of a comparison with a specialist registry. *Comm. Med.*, **11**, 81.

ALEXANDER, F.E., WILLIAMS, J., McKINNEY, P.A., RICKETTS, T.J. & CARTWRIGHT, R.A. (1989*b*). A specialist leukaemia lymphoma registry in the UK. Part 2: clustering of Hodgkin's disease. *Br. J. Cancer*, **60**, 948.

BARNES, N., CARTWRIGHT, R.A., O'BRIEN, C., RICHARD, I.D.G., ROBERTS, B. & BIRD, C.C. (1986). Rising incidence of lymphoid malignancies – true or false? *Br. J. Cancer*, **53**, 393.

BARNES, N., CARTWRIGHT, R.A., O'BRIEN, C. *et al.* (1987*a*). Variation in lymphoma incidence within Yorkshire Health Region. *Br. J. Cancer*, **55**, 81.

BARNES, N., CARTWRIGHT, R.A., O'BRIEN, C. *et al.* (1987*b*). Spatial patterns in electoral wards with high lymphoma incidence in Yorkshire Health Region. *Br. J. Cancer*, **56**, 169.

BLACK, SIR DOUGLAS (1984). *Investigation of the Possible Increased Incidence of Cancer in West Cumbria.* Report of the Independent Advisory Group. HMSO: London.

BOWIE, C. (1987). The validity of a cancer register in leukaemia epidemiology. *Comm. Med.*, **9**, 152.

BUCSKY, P. (1987). Hodgkin's disease: the Sternberg-Reed cell. *Blut*, **55**, 413.

CABANILLAS, F. (1988). A review and interpretation of cytogenetic abnormalities identified in Hodgkin's Disease. *Haematol. Oncol.*, **6**, 271.

CORREA, P. & O'CONNOR, G.T. (1971). Epidemiologic patterns of Hodgkin's disease. *Int. J. Cancer*, **8**, 192.

DRAPER, G.D., BIRCH, J.M., BITHELL, J.F. & 6 others (1982). *Childhood Cancer in Britian; Incidence Survival and Mortality.* Studies on Medical and Population Subjects, no. 37. HMSO: London.

DREXLER, H.G. & LEBER, B.F. (1988). The nature of the Hodgkin cell. *Blut*, **56**, 135.

FROME, E.L. (1983). The analysis of rates using Poisson regression models. *Biometrics*, **39**, 665.

GARDNER, M.J. & WINTER, P.D. (1984). Mortality in Cumberland during 1959–68 with reference to cancer in young people around Windscale. *Lancet*, **ii**, 216.

GLASER, S.L. (1986). Recent incidence and secular trends in Hodgkin's disease and its histologic subtypes. *J. Chron. Dis.*, **39**, 789.

GLASER, S.L. (1987). Regional variation in Hodgkin's disease incidence by histologic subtype in the US. *Cancer*, **60**, 2841.

GRIESSER, H., FELLER, A.C., MAK, T.W. & LENNERT, K., (1987). Clonal rearrangements of T-cell receptor and immunoglobulin genes and immunophenotypic antigen expression in different subclasses of Hodgkin's disease. *Int. J. Cancer*, **40**, 157.

HARRINGTON, D.S., YULING, Y.E., WEISENBURGER, D.D. & 4 others (1987). Malignant lymphoma in Nebraska and Guangzhou, China: a comparative study. *Hum. Pathol.*, **18**, 924.

KRISTOFFERSSON, U., HEIM, S., MANDAHL, N., OLSSON, H., AKERMANN, M. & MITELMAN, F. (1987). Cytogenetic studies in Hodgkin's disease. *Acta Pathol. Microbiol. Immunol. Scand.*, **95**, 289.

LEUKAEMIA RESEARCH FUND 1984 DATA COLLECTION GROUP (1987). Distribution of leukaemia, lymphoma and allied disease in parts of Great Britain: analysis by administrative districts and simulations of adjacencies. *Leukaemia*, **1**, 78.

LEVY, L.M. (1988). Hodgkin's disease in black Zimbabweans. *Cancer*, **61**, 189.

LUKES, R.J. & BUTLER, J.J. (1966). The pathology and nomenclature of Hodgkin's disease. *Cancer Res.*, **26**, 1063.

MACMAHON, B. (1966). Epidemiology of Hodgkin's disease. *Cancer Res.*, **26**, 1189.

MUIR, C., WATERHOUSE, J., MEEK, T., POWELL, J. & WHELAN, S., eds (1987). *Cancer Incidence in Five Continents, Volume 5.* IARC Scientific Publication no. 88. IARC: Lyon.

OPCS; (1978–1988). *Cancer Statistcs Registrations and Cases of Diagnosed Cancer Registered in England and Wales 1968–1980.* Series MB1, nos 1–16. HMSO: London.

STEIN, H., HAUSMANN, M.L., LENNERT, K., BRANDTZAEG, P., GATTER, K.C. & MASON, D.Y. (1986). Reed-Sternberg and Hodgkin cells in lymphocyte-predominant Hodgkin's disease of nodular type contain J chain. *Am. J. Clin. Pathol.*, **86**, 292.

STILLER, C.A. (1985). Descriptive epidemiology of childhood leukaemia and lymphoma in Great Britain. *Leukaemia Res.*, **9**, 671.

VAN HOFF, J., SCHYMURA, M.J., & McCREA, M.G. (1988). Trends in the incidence of childhood and adolescent cancer in Connecticut 1935–1979. *Med. Paediatr. Oncol.*, **16**, 78.