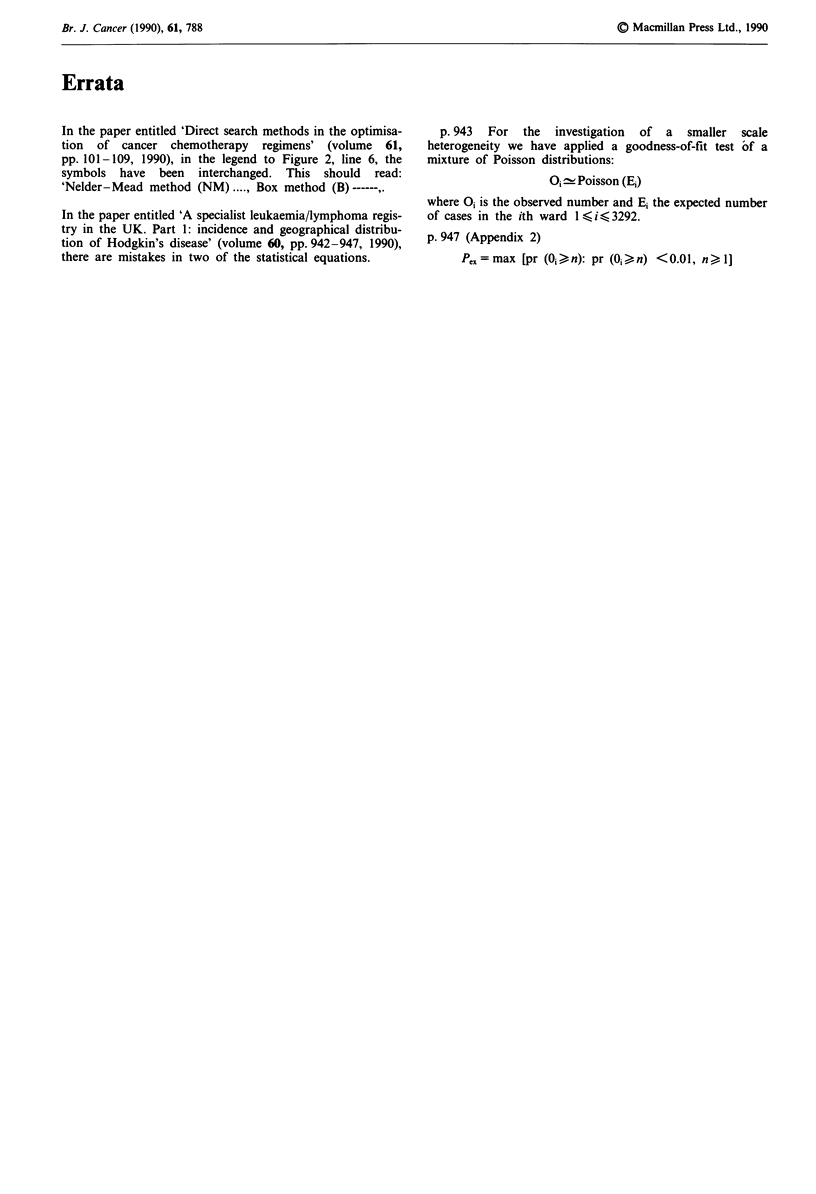# A specialist leukaemia registry

**Published:** 1990-05

**Authors:** 


In the paper entitled 'A specialist leukaemia/lymphoma registry in the UK. Part 1: incidence and geographical distribution of Hodgkin's disease' (volume **60**, pp. 942–947, 1990), there are mistakes in two of the statistical equations.

p. 943 For the investigation of a smaller scale heterogeneity we have applied a goodness-of-fit test of a mixture of Poisson distributions:

$$O_i \simeq \text{Poisson}\,(E_i)$$

where $O_i$ is the observed number and $E_i$ the expected number of cases in the $i$th ward $1 \leqslant i \leqslant 3292$.

p. 947 (Appendix 2)